# Trends in Nutrition-Related Risk Factors Identified Post-Operatively in Patients Treated for a Lower-Extremity Injury

**DOI:** 10.3390/nu16121847

**Published:** 2024-06-13

**Authors:** Caroline Podvin, Taylor Morrison, Jessica Dabis, James J. McGinley, Henry B. Ellis, Philip L. Wilson, Sophia Ulman

**Affiliations:** 1 Center for Excellence in Sports Medicine, Scottish Rite for Children, 5700 Dallas Parkway, Frisco, TX 75034, USA; ccpodvin@gmail.com (C.P.); taylor.morrison@tsrh.org (T.M.); jessica.dabis@tsrh.org (J.D.); jamesmcginley@att.net (J.J.M.); henry.ellis@tsrh.org (H.B.E.); philip.wilson@tsrh.org (P.L.W.); 2 Department of Orthopaedic Surgery, University of Texas Southwestern Medical Center, 1801 Inwood Road, Dallas, TX 75390, USA

**Keywords:** nutrition assessment, diet, youth sports, athlete, adolescent, risk factors, post-operative care, nutrition behaviors

## Abstract

This study investigated trends within a custom Sports Nutrition Assessment for Consultation (SNAC) survey designed to identify nutrition-related risk factors among post-operative lower-extremity youth athletes. Athletes aged 8–18 years who completed the SNAC at a sports medicine institution after lower-extremity surgery were reviewed for associations between SNAC questions and age/sex differences. Of 477 patients (15.0 ± 2.0 years; 47.8% female), 319 (66.9%) answered ‘yes’ to at least one question and were identified for a consult, though 216 (64.3%) declined. The most frequent questions to prompt a consult were a desire to better understand nutrition for recovery (41.5%) and regularly skipping at least one meal a day (29.8%). Inter-question responses were often significantly related, especially regarding appetite changes, weight changes, and/or meal-skipping. While consult acceptance was not significantly different between sex/age, males were more likely to report a desire to better understand nutrition (*p* = 0.004) and a weight change (*p* = 0.019), and females were more likely to report struggling with dizziness/fatigue (*p* < 0.001). Additionally, older athletes reported an appetite change (*p* = 0.022), meal-skipping (*p* = 0.002), a desire to better understand nutrition (*p* < 0.001), a weight change (*p* < 0.001), and an effort/recommendation to change their body composition/weight (*p* = 0.005). These findings demonstrate a link between risky nutrition behaviors and physical detriments.

## 1. Introduction

In the United States, nearly 27 million youths aged 6 to 17 years participate in sports and athletic activities [1]. Across recent years, participation trends demonstrate that youth athletes compete on multiple teams, train year-round, and specialize in sports at an early age, creating a highly competitive and demanding athletic environment [1,2,3]. This high level of participation underscores the importance of addressing the unique nutritional needs of this population. In addition to the energy demand required of daily activity, training, and competition, the typical youth athlete faces a unique stage of development when their body also requires energy to meet the demands of growth [4]. It is known that adolescence is a period of heightened nutritional demands due to growth and athletic activities combined with the unhealthy eating patterns and risky behaviors that occur in competitive athletics and the nutrition knowledge gap of this population [5]. Specifically, behaviors of adolescent athletes may include skipping meals, restrictive eating, over-exercising, and increasing dehydration risk with restriction of fluid intake, use of diuretics and saunas, and/or wearing nonporous suits to drastically increase production of sweat [6,7]. Furthermore, adolescent athletes often utilize nutritional supplements with the intention of improving athletic performance [8]. Those who participate in sports that emphasize body composition as a direct predictor of performance, such as leanness in distance runners, gymnasts, and dancers, face an especially high occurrence of disordered eating behaviors and attitudes in addition to body dissatisfaction [4,5,6].

While these behaviors can negatively influence athletic performance, they become increasingly harmful to the developing athlete who has sustained an injury. An optimal, balanced diet is extremely important to an athlete’s recovery and rehabilitation. Specifically, the recent literature has emphasized the importance of high-quality proteins, fat, vitamins, antioxidants, minerals, and other supplements during exercise-based rehabilitation to support athletes’ anabolism [9]. The essential role of energy and macronutrients requirements has been extensively investigated, indicating that athletes in recovery require high consumptions of energy via adequate carbohydrates and proteins [10]. Nutrition plays a crucial role in fighting muscle atrophy and anabolic resistance, managing inflammation and providing the body with adequate energy to heal [5]. Thus, it is critical to work with an athlete on their eating attitudes and behaviors during post-injury recovery. Improving nutrition habits can support the healing process, physical therapy efforts, and performance for recovered athletes returning to sport [10]. Thus, a Sports Nutrition Assessment for Consultation (SNAC) survey designed for injured youth athletes facing recovery may facilitate appropriate and effective interventions to promote their healing and support their futures as competitive athletes.

Numerous articles have recently published nutrition recommendations for youth athletes postoperatively [10,11,12,13], recent work has investigated sex and age differences in objective, nutrition-related measures (i.e., BMI, body composition) immediately postoperative [14,15], and one recent article was found validating a postoperative nutrition assessment of energy intake for adolescent athletes [16], but to our knowledge, there is no evidence that addresses the overall population of youth athletes and their nutrition-related risk factors immediately following surgical treatment. This is likely because no validated tool is currently available to screen patients for nutrition-related risk factors (i.e., attitudes, behaviors, and experiences) as they face post-operative recovery beyond provider discretion. The purpose of this study was to investigate trends within the responses of a custom sports nutrition assessment in the interest of better identifying nutrition-related risk factors among a post-operative lower-extremity patient cohort. As each question of the SNAC was designed to identify a different potential risk factor in nutrition-specific behaviors, it is important for clinicians employing the SNAC in clinic to understand how certain risky behaviors relate to others. For example, if a patient has experienced a recent change in weight, they may have recently started skipping meals or perhaps they are also feeling dizzy or fatigued at practice. While these behaviors may be intuitively related, the current study aims to highlight evidence-based associations. Additional areas of interest were age- and sex-related differences in SNAC responses. It was hypothesized that the SNAC would identify relationships between nutrition-related risk factors in a post-operative youth athlete cohort and that age- and sex-related differences would exist. Specifically, nutrition-related behaviors, such as skipping meals or aiming to change body composition, were anticipated to be strongly associated with poor sport experiences, including a history of stress fractures or experiencing dizziness or fatigue during training. Additionally, it was hypothesized that older female athletes would report worse nutrition-related risk factors. 

## 2. Methods 

### 2.1. Participants 

The current study received institutional review board approval with a waiver of informed consent before initiation of study procedures. Specifically, informed consent from patients was waived as all data collected and analyzed for the current study was captured as standard-of-care (i.e., the nutrition assessment is provided in clinic to all sports medicine patients post-operative). Thus, the current analysis was determined by the institutional review board to not expose patients to a greater than minimal risk and not adversely affect patients’ rights and welfare. A retrospective review of patients who underwent lower-extremity surgery at a single sports medicine treatment center was performed. Inclusion criteria were patients aged 8–18 years old, evaluated between 30 November 2021 and 19 January 2023 at their first post-operative clinic visit. No patients were excluded due to missing data. 

### 2.2. Procedures

Patient-specific information was assessed through patient-reported electronic intake questionnaires that were administered at clinic visits as standard of care. Demographic variables were captured in addition to menstrual period dates for female respondents and specific SNAC responses. 

An experienced sports medicine dietitian, in collaboration with other sports medicine medical staff, designed a custom sports nutrition assessment to identify potential nutrition-related risk factors immediately following surgery in a youth athlete population. The SNAC is a 9 to 14-question survey with ‘yes’ and ‘no’ response options (Table 1). The number of questions a participant receives depends on their sex, as females receive more questions than males regarding status of menarche. Each question of the SNAC investigates a different aspect of nutrition such as nutrition habits and preferences, fueling behaviors, goals, weight changes, and medical history of illnesses or symptoms related to under fueling. While most nutrition assessments in the recent literature focus on consumption (contents and frequency) [17,18] and/or nutritional knowledge [17,19,20,21], the primary goal in developing the SNAC was to identify risky behaviors, and/or experiences or feelings that may lead to risky behaviors, that may be mitigated through intervention via a consultation with a sports medicine dietitian. In addition to the influence of consumption- and knowledge-focused questionnaires, some questions are also based on a well-established nutrition risk screen developed for seniors [22] but have been adapted for the youth sport context. 

At the first post-operative clinic visit, the SNAC was completed electronically either by the patient or a parent. Answering ‘yes’ to any of the SNAC questions prompted a question asking the patient/parent if they would like a consultation with a sports dietitian. Additionally, female patients who responded ‘yes’ to missing a menstrual cycle for 3 months or more and those female patients who were 15 years or older and said they had not yet started their menstrual cycle were also prompted with the consultation question. If the patient chose ‘no’ to consulting with the sports dietitian, they were prompted to select why they chose against the consultation.

### 2.3. Statistical Analysis

Descriptive statistics (means, standard deviations, and frequencies) were computed for each variable. Fisher’s exact tests were used to determine whether nonrandom associations existed between the binary variables collected. Comparisons included responses between SNAC questions and sex differences in response to the SNAC. For each comparative test between SNAC questions, odds ratios were also computed to quantify the strength of the association between positive responses for each question. Independent samples *t*-tests were performed to determine age-related differences across binary variables (sex, SNAC questions, response to a consultation). Significance level (*α*) was set to 0.05, and no data were missing from the standard eight nutrition questions. Statistical analysis was performed using SPSS (IBM Corp., Armonk, NY, USA, https://www.ibm.com/spss). 

## 3. Results 

In total, 477 patients (15.0 ± 2.0 years) completed the SNAC of which 228 (47.8%) were female. Of the female patients, 209 (92.5%) reported having had a period, and 17 (8.5%) reported having recently ‘gone longer than 3 months without a period’. Across all respondents, 319 (66.9%) answered ‘yes’ to at least one question on the SNAC, indicating a considerable proportion of patients exhibited a nutrition-related risk factor. The most common positive responses indicated a popular desire to better understand nutrition for recovery purposes (198, 41.5%), followed by whether the patient regularly skipped at least one meal a day (142, 29.8%; Table 1). In contrast, the majority of respondents indicated that they did not have a history of stress fractures (456, 95.6%), struggle with dizziness or fatigue during training (441, 92.5%), or have any food allergies or intolerances (434, 91.0%). Of the 336 (68.2%) that were asked about their interest in a sports dietitian consult, 216 (64.3%) declined, primarily reporting that they did not feel as though it applied to them (Table 2). 

Furthermore, responses between questions were found to be significantly related (Table 3), especially for questions focusing on a change in appetite, change in weight, and/or skipping meals. Notably, minimal associations were identified with reporting a positive response to having food allergies/intolerances, a history of stress fractures, or a struggle with dizziness or fatigue during training. Specifically, respondents reporting a recent change in weight were 2.18 times more likely to report food allergies or intolerances (*p* = 0.042). Respondents reporting a history of stress fractures were 2.96 and 2.80 times more likely to indicate a desire to better understand nutrition for recovery purposes (*p* = 0.016) and a recent change in weight (*p* = 0.045), respectively. Respondents who indicated struggling with dizziness or fatigue during games, practices, or with exercise were 3.52 times more likely to report a recent change in appetite (*p* = 0.002), 2.89 times more likely to regularly skip at least one meal a day (*p* = 0.002), and 3.10 times more likely to be trying to or receiving a recommendation to change their body composition or weight (*p* = 0.003). Lastly, respondents were significantly more likely to express an interest in a consultation if they also indicated a desire to better understand nutrition (OR: 5.00, *p* < 0.001), experienced a recent change in weight (OR: 2.96, *p* < 0.001), or were trying to or received a recommendation to change their body composition or weight (OR: 1.65, *p* = 0.040). 

### 3.1. Sex Comparison 

**Figure 1:**
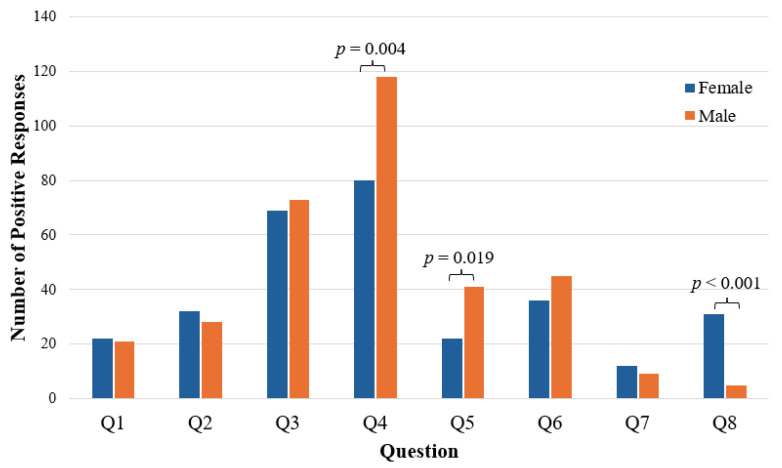
Sex differences in SNAC responses.

A total of 149 females (65.4% of females) and 170 males (68.3% of males) were prompted about a potential consult (Table 2). While the frequency of positive responses to the consult inquiry was not significantly different between sexes (*p* = 0.422), males and females did significantly differ in their response to individual questions of the SNAC (Table 1 and Figure 1). Specifically, males were more likely to report that they wished they had a better understanding of their nutrition for their recovery (*p* = 0.004) and that they had experienced a recent change in their weight (*p* = 0.019). Alternatively, females were more likely to report struggling with dizziness or fatigue during training (*p* < 0.001). 

### 3.2. Age Comparison 

No significant difference in age was identified by sex (*p* = 0.292). However, female respondents who indicated having had a period were significantly older (Yes: 209, 15.2 ± 1.7 years; No: 17, 12.8 ± 1.7 years; *p* < 0.001). Regarding the SNAC responses, age-related differences were found among five of the eight questions (Table 4). Significantly older respondents reported a recent change in appetite (*p* = 0.022), regularly skipping at least one meal a day (*p* = 0.002), a desire to better understand nutrition for their recovery (*p* < 0.001), a recent change in weight (*p* < 0.001), and trying to or receiving a recommendation to change their body composition or weight (*p* = 0.005). However, it is important to note that the greatest mean difference in age was only 0.82 years. 

## 4. Discussion

The purpose of the current study was to investigate trends among responses of a custom Sports Nutrition Assessment for Consultation (SNAC) survey created to indicate potential nutrition-related risk factors in a lower-extremity patient cohort post-operatively. While an emphasis on nutrition after sports surgery is addressed in the recent literature [14,15], the current study contains the only questionnaire created to identify specific habits which are risk factors for post-operative recovery to our knowledge. The identification of specific risk factors may assist in understanding which athletes are at risk of delayed recovery or reinjury. While post-operative weight, sex, age, and other risk factors are also valuable, some are non-modifiable and others may be directly reflective of a recent surgery (limited ability to eat, etc.). Conversely, the SNAC assesses modifiable risk factors which are likely more reflective of pre-surgical attitudes. The primary reasons patients were identified for a nutrition consult were that they (1) wished they better understood nutrition for recovery, (2) skipped at least one meal a day, or (3) were trying to or someone recommended they change their weight or body composition. These results support the recent literature investigating the lack of nutrition knowledge in adolescent athletes. A 2020 study found that young athletes lack key knowledge specifically related to Daily Recommended Intakes and supplements, or when they do have such knowledge, it comes from “unqualified or non-professional sources” [23]. This lack of knowledge has been further reported on in a review by Brown et al. (2021) [24], which emphasized that acquiring accurate food and nutrition knowledge could improve individuals’ ability to make healthy choices and build optimal dietary habits.

As 67% of athletes were identified for a consult but 64% declined, most often due to feeling as though it did not apply to them (57%), more responsibility rests with the clinician to coach proper post-operative nutrition. The feeling of athletes that the consult does not apply to them may be due to a belief that the athlete can handle their rehabilitation independently, potential embarrassment, or the determination that their positive response does not actually indicate poor nutrition. As clinicians, greater care to explain the normalcy of nutrition difficulties during rehabilitation may assuage some athlete concerns, and telehealth options may improve the burden on those concerned about time or cost. While some positive responses may indicate normal development, such as recent weight changes after a growth spurt, clinicians must look for responses which cannot be explained by a patient history and suggest further counseling. Athletes will benefit from meeting with a registered dietitian or receiving educational handouts/videos, if a dietitian is unavailable, to enable the athlete to optimize their nutrition in the way most needed for their return to sport. The SNAC has the potential to flag athletes struggling with disordered eating behaviors and indicate to a clinician which athletes put their current recovery at risk. 

### 4.1. Weight Concerns

Young athletes feel pressure to meet certain standards set by sport culture, society, coaches, parents, teammates or themselves. The positive responses to questions regarding a “recent change in weight” (13%) and “trying to or someone recommended I change my body weight or body composition” (17%) indicate that young athletes are already regularly concerned with weight and body image. The past ten years have seen a rise in studies assessing social media [25,26] and other body image views [27,28,29] of children as parents and providers have witnessed the negative impact poor body image can have on athletic participation and mental wellness [30]. For example, Bird and Rushton (2020) stated that athletes, specifically those in weight-based or aesthetic sports, are at higher risk of feeling pressure to restrict their energy intake. While we traditionally associate these concerns with older athletes, studies have also found that childhood, specifically the preschool and elementary school age, is often the critical stage when body image attitudes develop [31]. In fact, one study found that 50% of children between the ages of 7 and 12 years old wished to be slimmer [32]. These findings are important for clinicians to understand as concerns about weight and body shape in the younger years could compromise psychological health throughout later stages of life and increase the individual’s risk for eating disorders, particularly during the vulnerable stage of recovery [32,33]. The current study primarily involved post-menarchal athletes, but future work may look at similar nutrition survey data in even younger athletes.

### 4.2. Appetite and Under Fueling

Responses regarding a recent change in appetite, recent change in weight, and skipping at least one meal a day were found to be significantly related with those experiencing weight changes having a 3.93 times greater odds of appetite changes and 2.27 times greater odds of skipping meals. Our findings suggest that the majority of young athletes surveyed in the current study have inconsistent eating patterns, often skipping meals (30%), which is similar to recent results published in other studies on child and adolescent eating habits [6,23,34]. Due to child and adolescent athletes’ higher energy and nutrient needs, making all meals and eating additional snacks throughout the day is necessary to support proper growth and development, prevent burnout, and protect against nutrition-related injuries [35,36]. However, adolescent eating patterns are often characterized by meal-skipping, frequent snacking, and a higher intake of fast food [4,8,24] which may lead to under fueling of important nutrients such as calcium, iron, magnesium, and iodine [37,38]. 

Though not intuitively surprising, respondents struggling with dizziness and fatigue were more likely to also report a recent change in appetite (3.52 times), skipping meals (2.89 times), and recommendations to change their body composition (3.10 times). Someone trying to change their weight (especially during the early recovery phase) might view skipping meals as a method for weight loss, which could lead to under fueling that materializes as dizziness and fatigue with activity. This is concerning as it can set up the athlete for poor physical therapy sessions, muscle atrophy, and delayed recovery. The relationship found between a reported history of stress fractures, a desire to better understand nutrition for recovery, and a reported recent change in weight likely represent a continuation of this same under fueling issue that, if not addressed, could delay an injured athlete’s recovery and return to sport.

### 4.3. Sex- and Age-Related Differences

Both sexes may benefit from a sports nutrition consult after injury as 65.4% of females and 68.3% of males were identified in this study, but the reasons for this consult may differ. Males were more likely to report a desire to better understand nutrition and a recent weight change, while females were more likely to report struggling with fatigue and dizziness with exercise. Females often tend to desire a “leaner silhouette”, while males often desire a “larger body” [33]. Clinical experience dictates that females’ desire to be thinner can often lead to complications of under fueling, such as dizziness and fatigue with exercise. This under fueling has been estimated at nearly 50% in female athletes aged 15–30 years [39,40]. Even when females meet traditional sports nutrition standards for essential ingredients, such standards tend to be based largely on male athlete needs and may not adequately guide female athletes to avoid the effects of low energy availability [40]. Additionally, while a portion of males desire to be thinner or “leaner”, many male youth athletes desire to be bigger and more muscular which can often lead to supplement use, confusion on the best ways to gain weight and build muscle, and a desire to learn more about nutrition—which was assessed on the SNAC. Though the SNAC did not assess the direction of weight change, it is possible that weight changes represent an outcome of this desire for education on strength building. Alternatively, it may be that male athletes are less accustomed to calorie restriction, and during the immediate post-operative period at which they were assessed, males found themselves with undesired weight gain and more interest in learning about how to address it from a dietitian.

The data did not demonstrate age-related differences when comparing sex, but age-related differences were demonstrated in five of the eight SNAC questions with older respondents more frequently reporting a recent appetite change, skipping meals, a desire to understand nutrition, a recent weight change, and trying or being recommended to change body composition or weight. Athletes at older ages are more likely to participate in club- or national-level sport, which may both introduce them to poor nutrition habits which they believe to maximize their performance and provide them with an opportunity to work with team-sponsored dietitians. Similarly, they could be more motivated than the young, recreational athlete to learn about nutrition and take steps to alter their body composition, leading to the higher frequency of positive SNAC responses observed. Even in the absence of competition-level differences, older adolescents undergo a period of physical and psychological transformation during puberty which includes developmental weight and body composition changes as well as more awareness of the perception of others [41], likely reflected in the SNAC. Still, among these significant differences, athletes with positive responses tended to be only about eight months older, and younger children are not exempt from these risks. Thoughts and attitudes around weight have been established in children as young as three years old [31], and girls between the ages of five and seven years old with weight concerns have been more likely to show dietary restraint and other disordered eating behaviors, independent of their individual Body Mass Index [42]. Clinicians should, therefore, address nutrition with all patients post-operatively, taking extra care with those at older ages. An understanding of an athlete’s goals in life and in sport assists the clinician in tailoring the rehabilitation process to each child and providing appropriate dietary recommendations which correspond to their current and desired level of performance.

### 4.4. Limitations

The current study has certain limitations that should be noted apart from standard limitations associated with patient-reported outcomes which may be subject to under-reporting but also reflect the most feasible method of patient nutrition screening. Given that this study was conducted on athletes with lower-extremity injuries post-operatively at a single sports medicine clinic, the results presented here may not be generalizable to patients with a different injury profile or at another time point of recovery. Although not statistically evaluated given the uneven distributions, differences between races, ethnicities, and sports should be explored further in future studies as the relevant literature has indicated that nutrition behaviors in the general youth may differ based on these factors. Specifically, Lytle et al. reported that poorer diet habits were more commonly observed in African American and Hispanic youth compared to Caucasian students [43], and Neumark-Sztainer et al. found that non-White (i.e., African American, Hispanic, Asian American, and Native American) youth reported notably different weight-related concerns/behaviors [44]. Similarly, among different sports, various nutrition-related risks have been reported. Klein et al. reported that collegiate-level individual sport athletes were more knowledgeable about hydrations and micronutrients than team sport athletes [45], and numerous reports have highlighted sport-specific risk factors related to nutrition [46,47,48], such as with youth runners [48,49,50]. Targeted interventions such as broad testing of athletes at different private and public schools in varying demographics could better support such populations. 

Furthermore, the question investigating a recent change in appetite may have a relationship with the timeline of surgical intervention and potentially any pharmaceutical interventions administered post-operatively, which were not considered in the current analysis. Additionally, although this study did not investigate direction of weight gain and/or loss, future work could contribute to this knowledge gap by linking potential relationships between sex, age, and risk factors in nutrition to direction of weight change. A better understanding of these trends (e.g., females or younger athletes may drop considerable weight immediately post-operative compared to their male and/or older counterparts) would help clinicians be more proactive in providing nutrition education or interventions to counteract poor habits or a negative impact on recovery. 

## 5. Conclusions

The long-term goal of this work is to provide the research and clinical community with a valid and reliable tool that will likely improve awareness of nutrition-related risk factors and intervention for youth athletes. There is an urgent need for early screening tools to facilitate prompt detection of disordered eating patterns as well as nutrition knowledge deficits and ensure timely management of risky nutritional behavior that could delay an athlete’s recovery. Overall, 67% of athletes were identified for a nutrition consultation under the criteria of this study. Interest in a nutrition consultation was strongly related to a desire to better understand nutrition, a recent weight change, and a recommendation to change body weight. The significant relationships within the SNAC questions demonstrate the links between nutritionally risky behavior and physical detriments due to under fueling, such as dizziness and/or stress fractures. Given a high overall decline rate toward interest in a sports dietitian consult, often citing that respondents felt it was not applicable to them, more responsibility is placed on the clinician to determine which responses indicate normal development and which responses require further conversation. Quick standard-of-care tools such as the SNAC may assist with screening patients, and clinicians should suggest that athletes who display risky nutrition behaviors meet with a registered dietitian to proactively reduce re-injury risk and improve overall wellness.

## Figures and Tables

**Table 1:** Frequency (N, %) of responses to the Sports Nutrition Assessment for Consultation (SNAC).

Question	All	Female	Male
1. Do you have any food allergies/intolerances or avoid any food groups?			
*Yes*	43 (9.0)	22 (9.6)	21 (8.4)
*No*	434 (91.0)	206 (90.4)	228 (91.6)
2. Have you experienced any recent changes in appetite?			
*Yes*	60 (12.6)	32 (14.0)	28 (11.2)
*No*	417 (87.4)	196 (86.0)	221 (88.8)
3. Do you regularly skip at least one a meal a day?			
*Yes*	142 (29.8)	69 (30.3)	73 (29.3)
*No*	335 (70.2)	159 (69.7)	176 (70.7)
4. Do you wish you better understood nutrition for your recovery?			
*Yes*	198 (41.5)	80 (35.1)	118 (47.4)
*No*	279 (58.5)	148 (64.9)	131 (52.6)
5. Have you experienced any recent intentional or unintentional changes in your weight?			
*Yes*	63 (13.2)	22 (9.6)	41 (16.5)
*No*	414 (86.8)	206 (90.4)	208 (83.5)
6. Are you trying or has someone recommended that you change your body composition or weight?			
*Yes*	81 (17.0)	36 (15.8)	45 (18.1)
*No*	396 (83.0)	192 (84.2)	204 (81.9)
7. Do you have a history of stress fractures?			
*Yes*	21 (4.4)	12 (5.3)	9 (3.6)
*No*	456 (95.6)	216 (94.7)	240 (96.4)
8. Do you struggle with dizziness or fatigue during games, practices or with exercise?			
*Yes*	36 (7.5)	31 (13.6)	5 (2.0)
*No*	441 (92.5)	197 (86.4)	244 (98.0)

**Table 2:** Frequency (N, %) of responses to a sports dietitian consult.

Question	All	Female	Male
To optimize your return to sport rehabilitation, would you like to meet with our sports dietitian? *(% of prompted)*			
*Yes*	103 (32.3)	45 (30.2)	58 (34.1)
*No*	216 (67.7)	104 (69.8)	112 (65.9)
Please list the reason(s) for choosing *not* to have a sports dietitian consult. *(% of declined)*			
*I don’t feel like this applies to me*	124 (57.4)	63 (60.6)	61 (54.4)
*Time*	31 (14.3)	13 (12.5)	18 (16.1)
*No Response*	28 (13.0)	12 (11.5)	16 (14.3)
*Other*	19 (8.8)	11 (10.6)	8 (7.1)
*Financial Concern*	11 (5.1)	4 (3.8)	7 (6.3)
*Transportation*	3 (1.4)	1 (1.0)	2 (1.8)

Note: ‘Other’ reasons for declining the prompted consult included being ‘in good shape,’ having the resources to research on their own, having ‘a good diet’, not wanting one, already having a dietitian and/or trainer, and not living close.

**Table 3:** Odds ratios per inter-question association for the Sports Nutrition Assessment for Consultation (SNAC) questions and consult inquiry.

	Q2	Q3	Q4	Q5	Q6	Q7	Q8	Consult?
**Q1:** Food allergies/intolerances	1.40	0.60	1.70	2.18	1.13	2.52	1.29	1.22
95% CI [lower, upper]	[0.59, 3.30]	[0.28, 1.28]	[0.71, 3.19]	[1.02, 4.68]	[0.50, 2.54]	[0.81, 7.85]	[0.43, 3.83]	[0.63, 2.37]
*p*-value	0.287	0.123	0.066	**0.042**	0.451	0.110	0.411	0.338
**Q2:** Changes in appetite		2.15	2.36	3.93	2.41	1.68	3.52	1.31
95% CI [lower, upper]		[1.24, 3.73]	[1.36, 4.10]	[2.10, 7.36]	[1.30, 4.45]	[0.55, 5.17]	[1.63, 7.59]	[0.73, 2.34]
*p*-value		**0.005**	**0.002**	**<0.001**	**0.005**	0.265	**0.002**	0.223
**Q3:** Skip meals			2.28	2.27	1.81	1.48	2.89	0.98
95% CI [lower, upper]			[1.53, 3.39]	[1.32, 3.90]	[1.10, 2.97]	[0.60, 3.65]	[1.45, 5.74]	[0.62, 1.56]
*p*-value			**<0.001**	**0.003**	**0.014**	0.265	**0.002**	0.516
**Q4:** Desire better understanding				5.63	4.30	2.96	1.85	5.00
95% CI [lower, upper]				[3.05, 10.40]	[2.56, 7.22]	[1.17, 7.47]	[0.93, 3.66]	[2.82, 8.86]
*p*-value				**<0.001**	**<0.001**	**0.016**	0.055	**<0.001**
**Q5:** Experienced weight changes					13.11	2.80	2.01	2.96
95% CI [lower, upper]					[7.23, 23.79]	[1.04, 7.51]	[0.87, 4.62]	[1.69, 5.21]
*p*-value					**<0.001**	**0.045**	0.086	**<0.001**
**Q6:** Goal/Rec. to change weight						1.56	3.10	1.65
95% CI [lower, upper]						[0.56, 4.39]	[1.50, 6.42]	[0.98, 2.78]
*p*-value						0.276	**0.003**	**0.040**
**Q7:** History of stress fractures							1.31	0.61
95% CI [lower, upper]							[0.29, 5.85]	[0.22, 1.70]
*p*-value							0.482	0.240
**Q8:** Struggle with dizziness/fatigue								0.75
95% CI [lower, upper]								[0.35, 1.61]
*p*-value								0.291

Note: Odds ratios, 95% confidence intervals, and *p*-values from Fisher’s exact tests are presented. Odds ratios greater than 1 indicate a positive association (increased likelihood of a ‘yes’ response), while ratios less than 1 indicate a negative association (increased likelihood of a ‘no’ response). Significant *p*-values are in bold.

**Table 4:** Age differences by the Sports Nutrition Assessment for Consultation (SNAC) question.

Variable	Yes	No	*p*-Value
Food allergies/intolerances	15.1 ± 2.1	15.0 ± 2.0	0.982
Changes in appetite	15.6 ± 1.8	15.0 ± 2.0	**0.022**
Skip meals	15.4 ± 1.7	14.9 ± 2.1	**0.002**
Desire for better understanding	15.5 ± 1.8	14.8 ± 2.1	**<0.001**
Experienced changes in weight	15.8 ± 1.6	14.9 ± 2.1	**<0.001**
Goal/Recommendation to change weight	15.6 ± 1.8	14.9 ± 2.0	**0.005**
History of stress fractures	15.7 ± 1.6	15.0 ± 2.0	0.066
Struggle with dizziness or fatigue	15.1 ± 1.5	15.0 ± 2.1	0.684
*Positive Response for a consult*	15.5 ± 1.7	15.2 ± 1.9	0.183

Note: All values reported as means ± SDs. Significant *p*-values are in bold.

## Data Availability

The raw data supporting the conclusions of this article will be made available by the authors on request. The data are not publicly available due to ethical reasons.
